# Scintillation and cherenkov photon counting detectors with analog silicon photomultipliers for TOF-PET

**DOI:** 10.1088/1361-6560/ad2125

**Published:** 2024-02-13

**Authors:** Joshua W Cates, Woon-Seng Choong, Erik Brubaker

**Affiliations:** 1 Lawrence Berkeley National Laboratory, Berkeley, CA, United States of America; 2 Sandia National Laboratories, Livermore, CA, United States of America

**Keywords:** photon counting detector, analog SiPMs, TOF-PET

## Abstract

*Objective.* Standard signal processing approaches for scintillation detectors in positron emission tomography (PET) derive accurate estimates for 511 keV photon time of interaction and energy imparted to the detection media from aggregate characteristics of electronic pulse shapes. The ultimate realization of a scintillation detector for PET is one that provides a unique timestamp and position for each detected scintillation photon. Detectors with these capabilities enable advanced concepts for three-dimensional (3D) position and time of interaction estimation with methods that exploit the spatiotemporal arrival time kinetics of individual scintillation photons. *Approach.* In this work, we show that taking into consideration the temporal photon emission density of a scintillator, the channel density of an analog silicon photomultiplier (SiPM) array, and employing fast electronic readout with digital signal processing, a detector that counts and timestamps scintillation photons can be realized. To demonstrate this approach, a prototype detector was constructed, comprising multichannel electronic readout for a bismuth germanate (BGO) scintillator coupled to an SiPM array. *Main Results.* In proof-of-concept measurements with this detector, we were able to count and provide unique timestamps for 66% of all optical photons, where the remaining 34% (two-or-more-photon pulses) are also independently counted, but each photon bunch shares a common timestamp. We show this detector concept can implement 3D positioning of 511 keV photon interactions and thereby enable corrections for time of interaction estimators. The detector achieved 17.6% energy resolution at 511 keV and 237 ± 10 ps full-width-at-half-maximum coincidence time resolution (CTR) (fast spectral component) versus a reference detector. We outline the methodology, readout, and approach for achieving this detector capability in first-ever, proof-of-concept measurements for scintillation photon counting detector with analog silicon photomultipliers. *Significance.* The presented detector concept is a promising design for large area, high sensitivity TOF-PET detector modules that can implement advanced event positioning and time of interaction estimators, which could push state-of-the-art performance.

## 1. Introduction

Time-of-flight positron emission tomography (TOF-PET) employs 511 keV photon interaction time in the PET detector ring to estimate the origin of annihilation photon pairs along system response lines drawn between two detector elements in coincidence. Annihilation event origins are constrained to normally distributed kernels with full-width-at-half-maximum (FWHM) dictated by the achievable coincidence time resolution (CTR) between detector element pairs. Incorporating TOF information into PET image reconstruction yields substantial gains in reconstructed image signal-to-noise-ratio (SNR) by localizing events close to their origin, rather than distributing counts across entire lines of response between detector elements, as is the case for standard PET reconstruction. The magnitude of this SNR gain scales with improved CTR. State-of-the-art clinical systems achieve approximately 200–400 ps CTR (Miller *et al* 2015, Hsu *et al* 2017, van Sluis *et al* 2019), enabling event localization between 3-6cm and providing an estimated 3.7–2.6-fold improvement in reconstructed image SNR (as calculated by estimated SNR gain from TOF technique in Conti (2008), relative to reconstruction with no TOF information incorporated. Ongoing research and development in this field aims to push CTR below 100 ps, towards the limit dictated by positron range profiles of ^18^F, at approximately 10 ps (Lecoq *et al* 2020).

In order to accomplish the ambitious task of realizing sub-100 ps FWHM CTR in large area, high sensitivity scintillation detector modules, each piece of the detection chain must be optimized. An ideal realization of a photosensor for scintillation detectors in TOF-PET would be one that can uniquely record the time-of-arrival of optical photons with high precision. Such a device would enable advanced time of interaction estimators and 3D interaction-dependent data corrections which fully leverage the intrinsic relationship between 511 keV position of interaction and spatiotemporal arrival time kinetics of scintillation light (van Dam *et al* 2013, Tabacchini *et al* 2015, Loignon-Houle *et al* 2021). This capability serves as a pathway for ultra-precise timing in high sensitivity (thick) detectors, where 3D position-of-interaction-dependent 511 keV photon and scintillation photon transit time jitter must be overcome. Moreover, 511 keV photon detection time can be derived from more advanced estimators than a simple average (i.e. leading edge time pickoff on a scintillation pulse), which may not be optimal for a given scintillation detector or for media leveraging prompt optical phenomena (Gundacker *et al* 2018, Loignon-Houle *et al* 2023).

Previous developments of single photon avalanche photodiode (SPAD) arrays and digital silicon photomultipliers (dSiPMs) aimed to achieve these capabilities in large area devices (Haemisch *et al* 2012, Mandai and Charbon 2013 as examples), where each Geiger-mode cell is latched, digitizing each detected photon. A comprehensive overview of these developments has been presented in Schaart *et al* (2016). In short, dSiPMs promise excellent single photon time resolution (SPTR) from single SPAD readout, fast recovery from active quenching, photon counting from the sum of digital triggers initiated from cell discharge, and multiple timestamps from individual pixels, as defined by the sensor's architecture. A collection of works with the Philips digital photon counting sensors (PDPCs) (van Dam *et al* 2013, Tabacchini *et al* 2015, Borghi *et al* 2016a, 2018) outlined techniques for leveraging first photon arrival time at pixels of the dSiPM array for maximum likelihood-based time of interaction estimators, which accounted for scintillation photon transit time in monolithic crystals. These studies demonstrated CTR commensurate with today's state-of-the-art commercial TOF-PET systems more than five years in advance with essentially half the photon detection efficiency (PDE) of SiPM arrays available today, highlighting the benefit of sensors and methods that exploit scintillation arrival time kinetics to derive estimates for 511 keV photon time of interaction that account for temporal variance in the detection chain. Promising efforts to develop advanced dSiPMs are in progress (Pratte *et al* 2021), and it seems likely that ideal dSiPMs (count and provide a timestamp for each optical photon) are available in the future. However, there may be alternative approaches to achieve these goals today.

In this work, we combine a monolithic scintillation crystal, optically coupled to an SiPM array, and low noise, high frequency electronic readout for a proof-of-concept demonstration of a scintillation photon counting detector concept, comprised entirely of off-the-shelf components. After presenting the methodology and conceptual basis for our scintillation photon counting detector, we outline an experimental setup designed for studies with the detector. We show the SPTR for the detector readout and experimental setup, demonstrate the scintillation photon counting capability of the detector with a monolithic bismuth germanate (BGO) scintillator, highlight the ability to implement 3D event positioning information for data corrections that improve time of interaction estimation, and quantify the energy and timing resolution of our prototype detector. In discussion and interpretation of our results, we also outline a tractable electronic readout topology to realize this detector concept in imaging systems. Altogether, the primary aim of this work is to show that scintillation photon counting detectors and the advances they can bring to PET imaging can be realized today.

## 2. Materials and experimental methods

### 2.1. Photon counting scintillation detector concept

**Figure 1.**
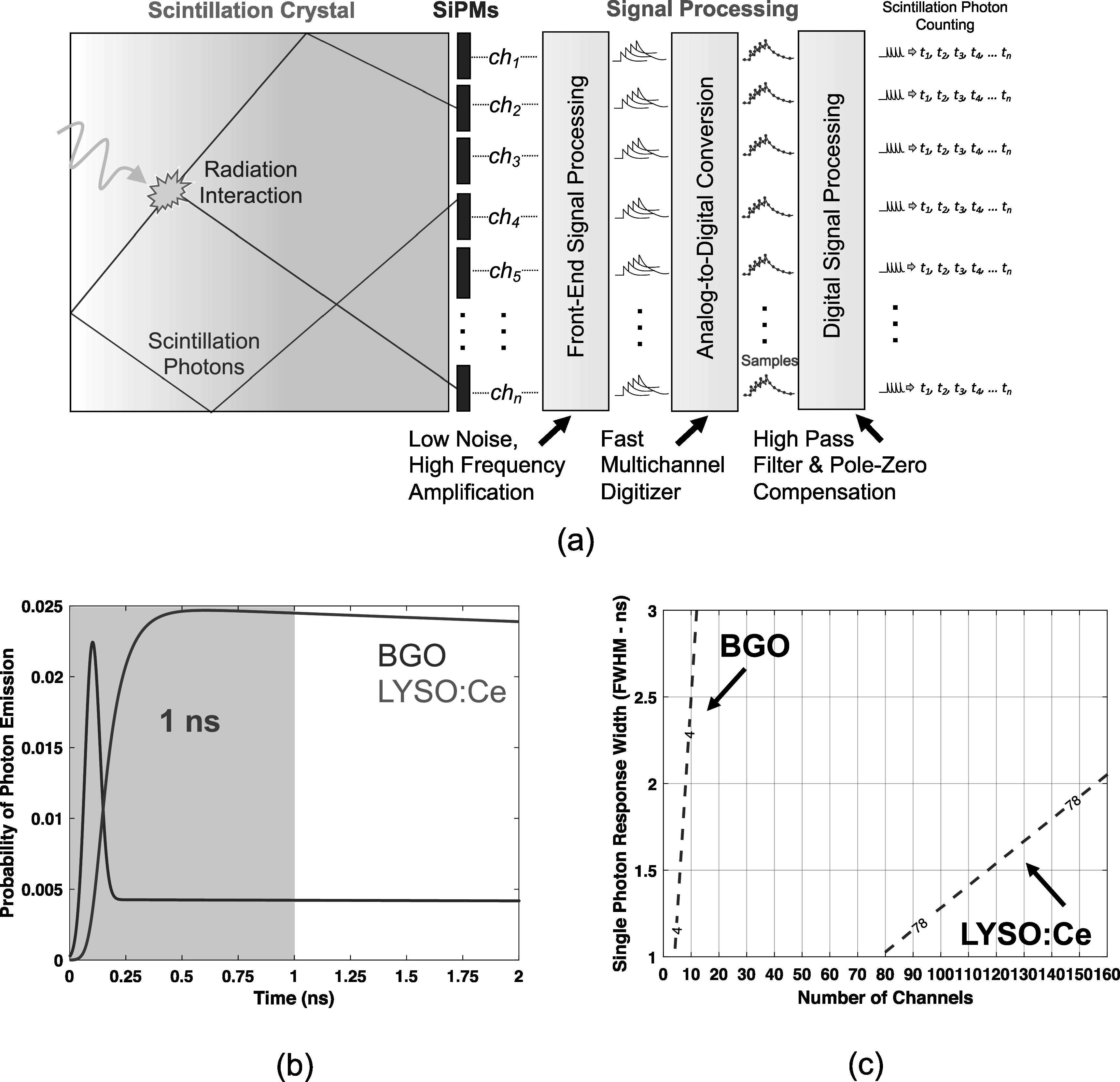
An illustration of a detector concept for counting photons from a scintillation detector with analog SiPMs is shown in (a). In (b), photon detection time profiles for BGO and LYSO:Ce scintillators are shown, highlighting that the highest temporal emission density occurs within the first nanosecond of excitation. An approximate calculation of single photon response shape width and number of channels required to count scintillation photons for BGO and LYSO:Ce with the detector concept (a) is shown in (c).

If photon arrival time density, photosensor channel density, performance of electronic readout, and width of detector single photon response shape are all taken into consideration, one can derive detector configurations that perform counting on streams of detected optical photons with minimal overlap, providing time pickoff and channel position for each photon. We illustrate a scintillation light detection processing chain for our idealized approach in figure 1(a), where each channel of an analog SiPM array has dedicated, high performance readout, and signals from each channel are digitized and shaped to provide single photon signatures with discrete amplitudes that can be counted. The basic concept is to spread scintillation light over a photosensor array in a scintillator monolith to create temporal sparsity in the arrival time profile of scintillation light at each channel, such that photons are separated in time by an amount greater than the FWHM of the instrument's single photon pulse shape. The concept outlined in figure 1(a) can be realized if sensor channel density and impulse response shape are appropriately matched with a scintillator’s luminosity. In realizing such a detector configuration, consider that the highest temporal emission density for scintillators commonly employed in TOF-PET detector research and development, for example LYSO:Ce and BGO, occurs within the first nanosecond (ns) after excitation, as highlighted in figure 1(b). Thus, if one could appropriately configure a monolithic scintillation detector with a large enough monolith and sufficient channel density to generate temporal sparsity in the arrival time profiles of scintillation photons within the first nanosecond, the detector would then also be capable of uniquely counting each optical photon in the remainder of the emissions envelope.

Considering the criteria outlined above, a first estimate of the number of sensor channels and single photon response width required to count photons arriving within the first nanosecond of interaction can be made for the scintillators listed in table 1, as shown in figure 1(c). For these calculations, photon detection time is derived from the convolution of the photon emission time profile, *f* ^*p*^(*t*∣*θ*) in equation (1), and the sensor's impulse response function, *g*(*t*) in equation (2), as outlined in Gundacker *et al* (2018). *f* ^*p*^(*t*∣*θ*) represents a probability density function over time *t* given scintillation start time *θ* for the scintillation emissions envelope dictated by the material's rise time, *τ*_*r*_, and decay time, *τ*_*d*_, for each component *i*, weighted by *ρ*_*i*_. The term *C*_*amp*_ *δ*(*θ*) is also included to incorporate prompt emissions, such as Cherenkov light, in a Dirac function, *δ*(*θ*), with amplitude *C*_*amp*_ (which is zero if no prompt emissions are present). The impulse response function, *g*(*t*), is dictated by the standard deviation of the timing uncertainty profile for a single photon detection, *σ*_*IRF*_, and also includes a term to account for electronic delay, Δ_*M*_. In these estimations, we assumed uniform light spread over the sensor array, light collection efficiency commensurate with 20mm thick crystal elements of each material and photon detection efficiency for a Broadcom AFBR-S4N33C013 SiPM operated at 7 V above breakdown voltage (*V*_br_) (Broadcom 2023). Numerical values along the dashed lines in figure 1(c) indicate the estimated number of detected optical photons within the first nanosecond. The impulse response width and number of channels required to achieve temporal sparsity are quite reasonable and could be achieved with moderately sized analog SiPM arrays with fast electronic readout. For example, a typical 4 × 4 SiPM array should be appropriate for BGO when an electronic readout achieves ≤4 ns FWHM impulse response width, and a13 × 13 array with ≤2 ns FWHM response shape could be sufficient for achieving optical sparsity with an LYSO:Ce monolith. We present these simple approximations to aid in explaining our proposed detector concept and provide a starting point for choosing a prototype electronic readout topology. Realizing this concept in a physical detector implies a more complicated relationship between 3D positions of interaction within the scintillator volume and achievable temporal sparsity in optical photon arrival time profiles.

\begin{eqnarray*}\begin{array}{c}{f}^{p}(t| \theta )={\mathrm{\Theta }}(t-\theta )\displaystyle \sum _{i=1}^{\infty }\frac{{e}^{\frac{-(t-\theta )}{{\tau }_{d,i}}}-{e}^{\frac{-(t-\theta )}{{\tau }_{r,i}}}}{{\tau }_{d,i}-{\tau }_{r,i}}\cdot {\rho }_{i}\,+\,{C}_{{amp}}\delta (t-\theta )\end{array}\end{eqnarray*}

\begin{eqnarray*}g(t)=\displaystyle \frac{1}{{\sigma }_{{IRF}}\sqrt{2\pi }}{e}^{\tfrac{{\left(t-{{\mathrm{\Delta }}}_{M}\right)}^{2}}{2{\sigma }_{{IRF}}^{2}}}\end{eqnarray*}

\begin{eqnarray*}{f}_{g}^{p}(t| \theta )={\int }_{-\infty }^{\infty }{f}^{p}({t}^{{\prime} }| \theta )g(t-{t}^{{\prime} }){{dt}}^{{\prime} }\end{eqnarray*}

**Table 1.** Photon emission, light collection efficiency (LCE) and PDE used to estimate the number of detected photons for example BGO and LYSO:Ce detectors.

Scintillation	Light Yield	Rise Time	Decay time	LCE	PDE^a^
Material	(photons/Mev)	(ps)	(ns)	(%)	(%)
BGO	10,200^c^; 17^b^ ^,^ ^c^	8^c^	45.8 (8%)^c^, 365 (92%)^c^	26	40^d^; 38^b^ ^,^ ^d^
LYSO:Ce	40,000^e^	77^f^	40^f^	39^g^	48^d^

^a^ SPTR of AFBR-S4N33C013 SiPMs reported from [Kratochwil *et al* 2021] (78 ps) and electronic delay assumed as 3× SPTR;

^b^ Cherenkov emissions related parameter;

^c^ [Gundacker *et al* 2020].

^d^ Data calculated from weighting photon emission spectra with PDE of AFBR-S4N33C013 SiPM at *V*_br_+7 V;

^e^ [Turtos *et al* 2016].

^f^ [Gundacker *et al* 2018].

^g^ [Gundacker *et al* 2014].

### 2.2. Experimental setup and prototype readout electronics

**Figure 2.**
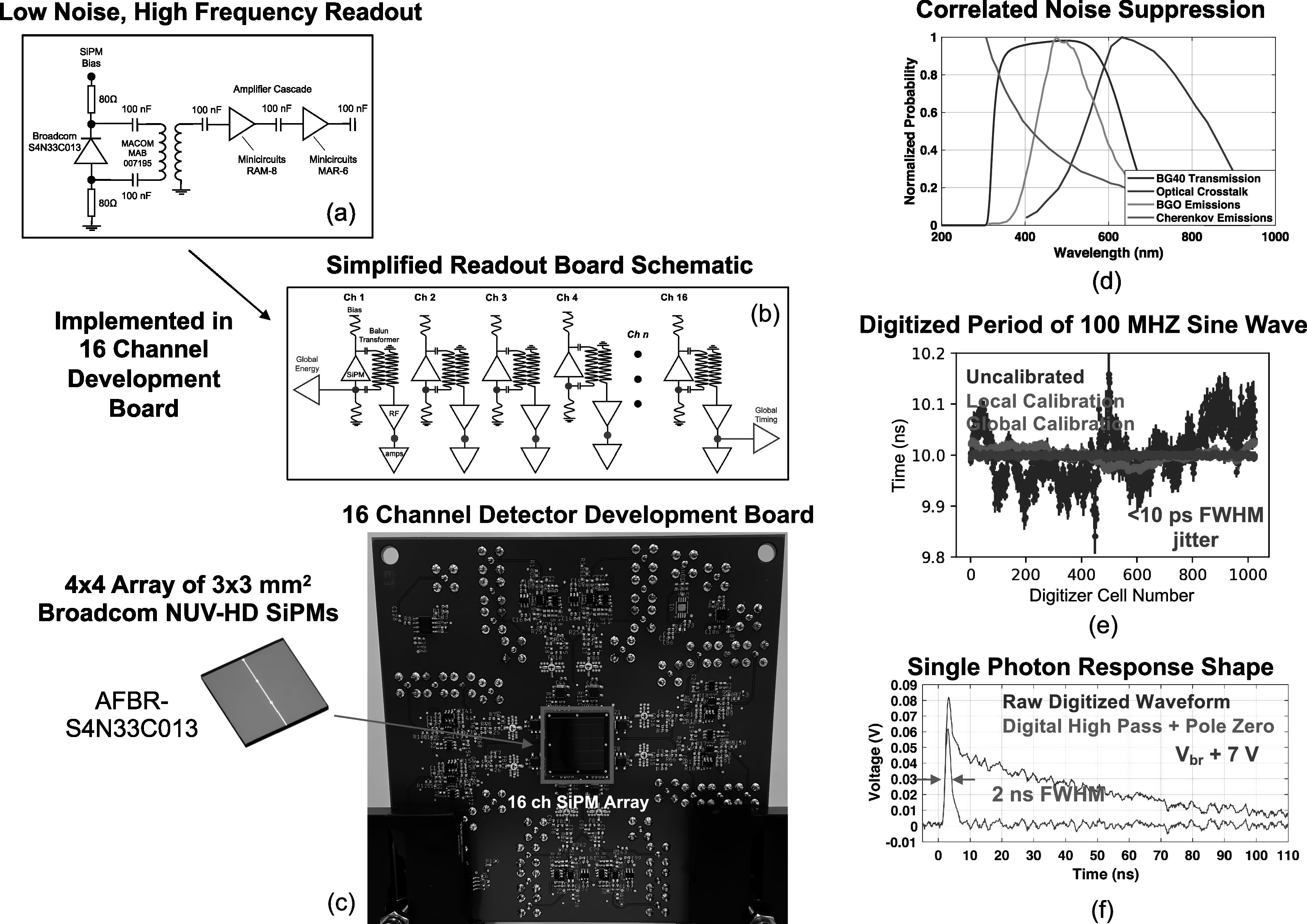
Key aspects of a prototype demonstration setup for a scintillation photon counting detector concept are shown. In (a), a simplified, single channel schematic is shown for a sixteen-channel prototype detector readout board. A simplified schematic of the prototype readout board is shown in (b), including multiplexed channels for data acquisition triggering. The sixteen-channel electronics board is shown in (c). Transmission plot for a Schott BG40 optical glass filter used for dramatically reducing external crosstalk from the SiPM array is shown in (d), along with the BGO, Cherenkov, and optical crosstalk emissions spectra. Each channel of data acquisition was custom calibrated to optimize digitization accuracy and intrinsic jitter of the experimental setup. In (e), the measured period of a 100 MHz sine wave, randomly phased between cells of a DRS4 chip channel is shown without and with calibrations applied. When calibrations are applied, the period is accurately quantified to <10 ps FWHM accuracy across the entire 200 ns time range. A measured single photon pulse from the prototype setup is shown in (f) with and without digital shaping applied (high pass filter with pole-zero compensation), achieving 2 ns FWHM pulse width.

Based on the approximation of channel number required to demonstrate our proposed photon counting detector concept, shown in figure 1(c), we designed a prototype demonstrator for a 12 × 12 × 15 mm^3^ BGO scintillator (Shanghai Project Crystal, Ltd) with mechanically polished surfaces, wrapped in Teflon tape. Using BGO for the prototype setup facilitated demonstration at a scale appropriate for a proof-of-concept study, requiring fewer channels of electronic readout. BGO has also received renewed interest for TOF-PET (Kwon *et al* 2016, Brunner and Schaart 2017, Cates and Levin 2019, Kratochwil *et al* 2020, Gundacker *et al* 2020, Kratochwil *et al* 2021, Gonzalez-Montoro *et al* 2022) due to its moderate Cherenkov yield in combination with the development of SiPMs having high PDE in the ultra-violet (UV) region and fast electronic readout that provides excellent SPTR (Cates *et al* 2018, Gundacker *et al* 2019). This crystal size was chosen to match the size of a custom, 4 × 4 array of 3 × 3 mm^2^ Broadcom AFBR-S4N33C013 SiPMs, as shown in figure 2. A custom, sixteen-channel electronics readout board was also designed which employed a modified version of the low noise, high frequency (LNHF) signal processing chain described in (Cates and Levin 2019). Signals from the detector readout were directly connected to sixteen channels of a CAEN V1742, DRS4 chip-based (Ritt 2008) digitizer, which digitized detector waveforms at 5 Giga-Samples-per-second (GSa/sec). A custom calibration of the digitizer was performed according to the methods outlined in (Kim *et al* 2014), to provide <10 ps FWHM intrinsic jitter (figure 2(d)). Digitized waveforms were processed with a simple high pass filter, followed by pole-zero compensation. The time constant for the high pass filter was parametrically varied, where the fastest time constant which could also be fully compensated was selected (3 ns). A digitized single photon pulse before and after digital pulse shaping is shown in figure 2(f). Ultimately, a 2 ns FWHM was achieved for single photon pluses.

A unique feature of our proposed detector configuration is the inclusion of an optical bandpass filter between the scintillator and SiPM array. A major issue for operating SiPM-based scintillation detectors at high overvoltage, which optimizes PDE and SPTR, is the generation of external optical crosstalk (Gola *et al* 2014). Optical photons generated from Geiger avalanche can be transmitted into the crystal volume, reflect at crystal boundaries, and be transported back to the SiPM. For a large SiPM array with electronic readout sensitive to single optical photons, coupled to a monolithic scintillator, this effect can dramatically impact SNR and achievable timing performance by limiting SiPM bias to lower operating voltages. Previous works have suggested and demonstrated the idea of filtering external optical crosstalk with an optical bandpass that is transmissive to scintillation light and absorptive to crosstalk photon emissions (Barton *et al* 2009, Masuda *et al* 2021). Here, we apply this concept, for the first time, to a monolithic scintillation detector. Figure 2(d) shows a transmission profile for a 1mm thick Schott BG40 optical glass filter, along with BGO scintillation light emission profile (Brunner and Schaart 2017), Cherenkov light profile (limited by the UV absorption edge in BGO), and emissions profile for optical crosstalk photons (Barton *et al* 2009). In this figure, the BG40 transmission probability is absolute, but the emissions profiles are normalized to the maximum value of each distribution, for clarity. Coupling an optical bandpass filter, such as BG40, between the BGO and SiPM array can thereby dramatically reduce external optical crosstalk with minimal impact on scintillation and Cherenkov emissions.

### 2.3. Single photon time resolution measurements

**Figure 3.**
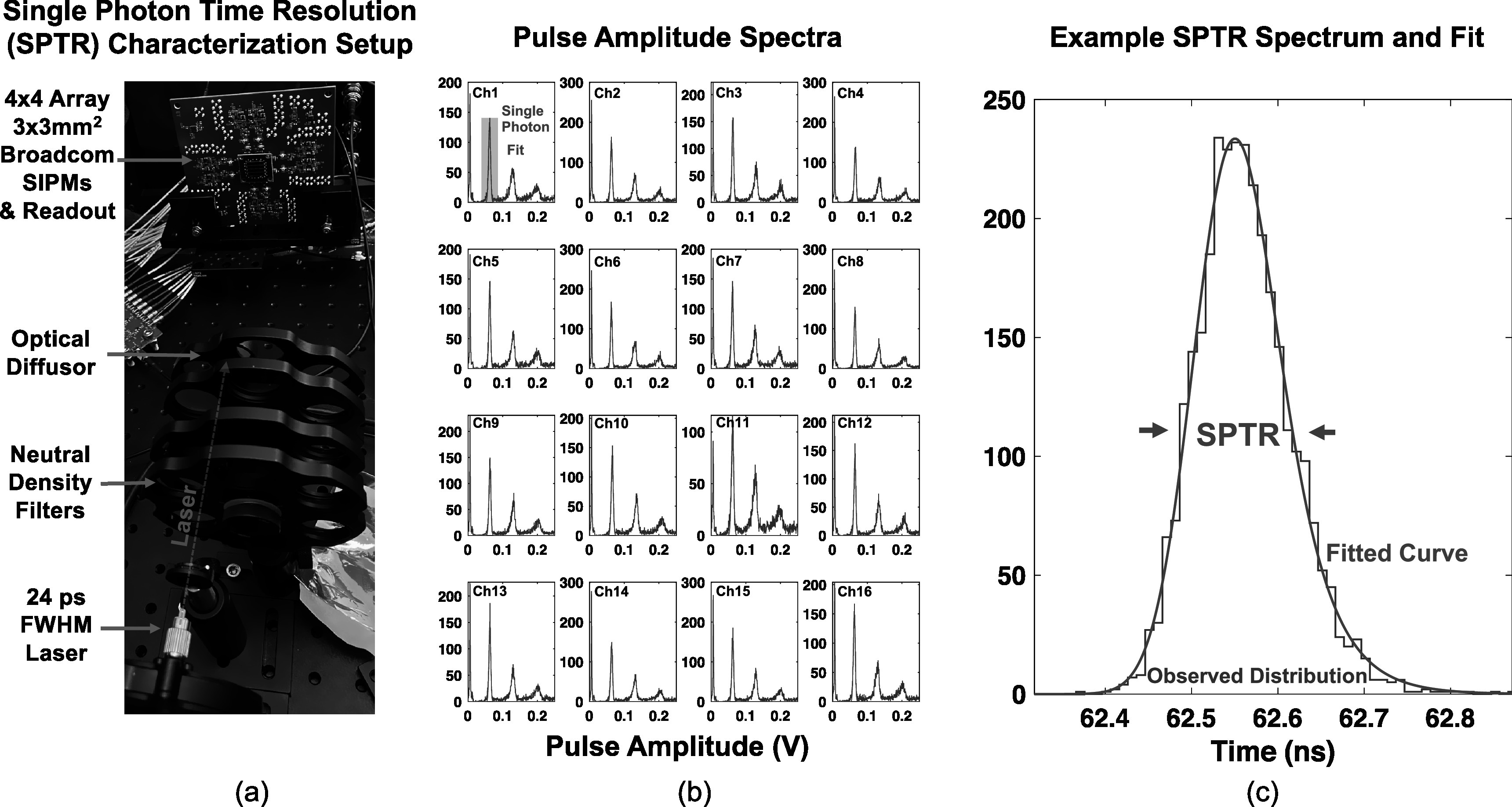
Experimental setup for characterization SPTR with the prototype detector readout is shown in (a). Single photon events were selected in post processing, as demonstrated in (b), and time difference distributions were built from the delay between a trigger provided by the pulsed laser and time pickoff on single photon pulses. Resulting distributions were fit with a combination Gaussian and exponential function, as illustrated in (c), where SPTR was taken from the FWHM of the fit.

SPTR of the prototype readout was quantified with the experimental setup shown in figure 3(a). Light from a PicoQuant laser (24 ps FWHM pulse width and 408 nm wavelength) was attenuated with a neutral density filter and evenly dispersed over the prototype readout's 4 × 4 SiPM array with an optical diffuser. An external trigger produced by the laser was used to trigger acquisition with the V1742 digitizer and also provide a ‘start’ time for the SPTR measurement. Twenty thousand waveforms were acquired for measurements with SiPMs biased at *V*_br_+5, 7, 9, 10, 11, and 12 V. Single photon events were selected from histograms of pulse amplitude (figure 3(b)), and waveforms for selected events were fit with a cubic spline, from which 10 ps trace sampling was produced. Time pickoff was performed using leading edge discrimination with a threshold set at half of the single photon amplitude. The resulting time difference spectra were fit with a Gaussian and exponential convolution (Nemallapudi *et al* 2016), as depicted in figure 3(c), where SPTR was taken from the FWHM of the resulting distribution.

### 2.4. Photon counting detector measurements

**Figure 4.**
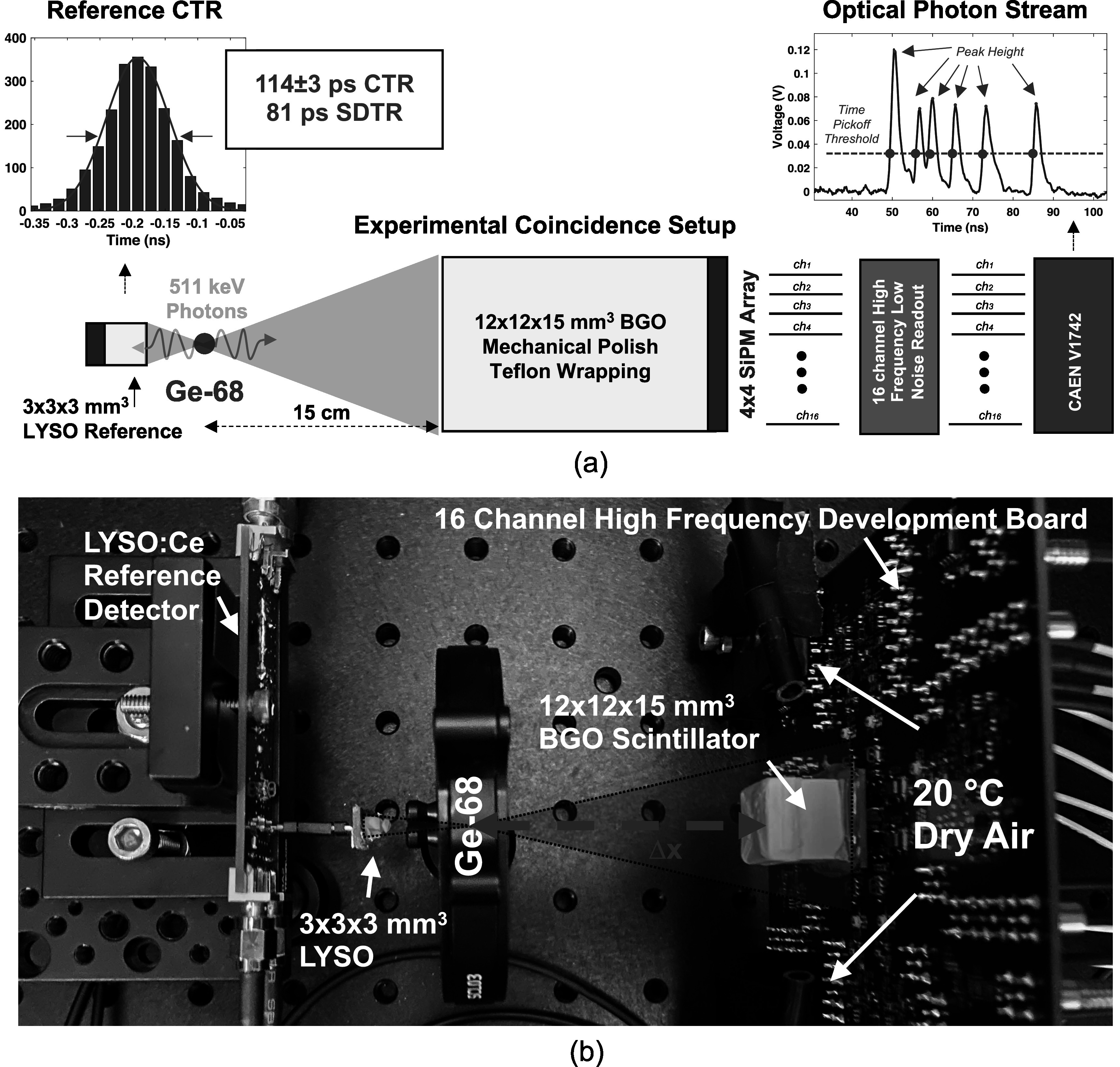
An illustration of the experimental setup for collecting coincidence data with the prototype detector and a small reference detector is shown in (a). A picture of the components of the setup is shown in (b).

Measurements to demonstrate and quantify the photon counting capability of the detector prototype were performed with the experimental setup shown in figure 4. The 12 × 12 × 15 mm^3^ BGO detector was integrated into a back-to-back coincidence measurement versus a 3 × 3 × 3 mm^3^ LYSO:Ce scintillator (Shanghai Project Crystal, Ltd.) optically coupled to a 3 × 3 mm^2^ AFBR-S4N33C013 SiPM with the same LNHF readout circuit employed in (Cates and Levin 2019). The timing and energy signals of the reference detector were also connected to separate channels in the V1742 digitizer. The energy signal from the reference detector and a global energy signal provided by the photon counting detector readout were fed to two channels of a constant fraction discriminator (CFD) module. CFD thresholds were adjusted such that they were just below the photopeak for the LYSO:Ce reference and BGO photon counting detector. CFD triggers were processed by a Philips Scientific 755 quad majority logic unit, which provided a coincidence logic pulse to trigger acquisition of the V1742 digitizer. The reference detector SiPM was operated at 38 V, where its timing performance was previously quantified to be 114 ± 3 ps FWHM CTR versus an identical reference detector in this setup, yielding 81 ps single detector time resolution (SDTR). The photon counting prototype's SiPM array was operated at 34 V (*V*_br_+7 V). This operating voltage was limited by the 1 V dynamic range of the digitizer. The LNHF electronic readout provides high gain and large amplitude for single photon pulses. If raw detector channel waveforms ‘clip’ at the top of the digitizer's dynamic range, then they cannot be digitally shaped properly, resulting in flat distortions that prohibit the experimental setup's ability to count optical photons. A 10 *μ*Ci Ge-68 source was placed between the two detectors, and 56 499 coincidence events were collected, producing 32 618 energy qualified coincidence events in both the reference and photon counting detector. The source and photon counting detector were placed 15 cm apart, to create virtually uniform irradiation across the area of the detector module. A picture showing major components of the experimental setup is shown in figure 4(b) (source and photon counting detector were spaced by 15 cm before data acquisition).

Photon counting was performed in offline analysis with a simple peak finding algorithm on digitally shaped traces from channels of the photon counting detector. The number of detected optical photons for each event was quantified by dividing the peak height of each optical photon pulse by the single photon pulse amplitudes recorded during the SPTR measurements, at the same overvoltage (figure 3(b)). Event energy was estimated by counting the total number of optical photons detected in each channel, for each event. Time pickoff was performed with an event validation scheme previously presented with BGO and the Philips digital photon counting detectors (Brunner and Schaart 2017). Timestamps from each optical photon voltage pulse, from each channel, were sorted into a single list. The first single photon detected, validated by the condition that at least 15 additional optical photons were detected within the following 10 ns, was chosen for time pickoff with a leading-edge discriminator, having a threshold set at half the single photon pulse height.

### 2.5. 3D positioning and data corrections

Relative 3D positioning of 511 keV photon interactions was also performed, where ‘x’ and ‘y’ coordinates were calculated from simple energy weighted mean positioning algorithm called ‘raise to a power’ (Pani *et al* 2016), where weights were squared in the calculation. Relative depth-of-interaction (DOI) was calculated from the sum of the squared number of photons at each pixel, which has previously been used for depth estimates in monolithic scintillators (Borghi *et al* 2016a). 3D position of 511 keV interactions was used to demonstrate corrections for energy and CTR. Note that collimated source calibrations to provide absolute positioning are beyond the scope of this manuscript. Here, we calculate a relative estimate for 3D position of interaction.

A position-dependent correction for energy measurement was performed by separating the crystal volume into 18 voxels (3 × 3 × 2 in the *x*, *y*, and depth directions, respectively), fitting the photopeak position in each voxel, and using the fitted means to align spectra before combining into a global energy spectrum. Since our relative 3D positioning for events is derived analytically, there is some inherent nonlinearity and bias across the detector volume. Thus, we have separated the crystal volume into relatively coarse ‘interaction voxels’ that segment the detector into ‘edge’ and ‘center’ regions, with two depth bins for each voxel.

**Figure 5.**
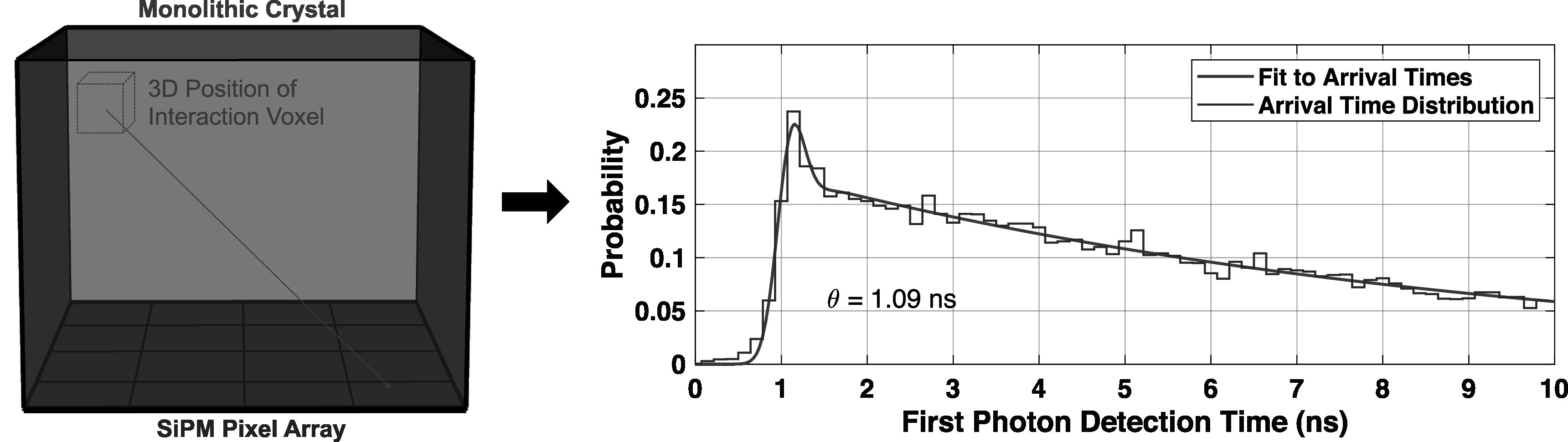
An example first detected photon distribution and with fit applied, according to equation (3), which was derived for each SiPM pixel, for each voxel of interaction.

A 3D position-dependent correction for optical photon time dispersion in the crystal was also performed by separating the crystal volume into 18 voxels. The first detected photon timestamp at each pixel, for each event, in each 3D segment, was used to create first photon detection probability distributions, for each SiPM pixel, as has previously been shown in van Dam *et al* (2013). These distributions create a correction for transit skew (511 keV photon transit, optical photon transit, and any electronics time skew) for each of the sixteen channels, for each detector voxel. The distributions were fit with equation (3). Figure 5 shows an example first detected photon arrival time distribution with corresponding curve fit. From each curve fit, the *θ* parameter was extracted to characterize first photon arrival time delay.

## 3. Results

### 3.1. Single photon time resolution

**Figure 6.**
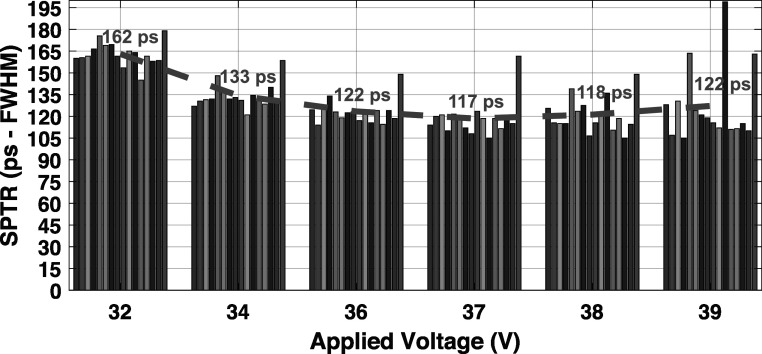
SPTR is plotted for sixteen channels of the detector module as a function of applied overvoltage. The average of all sixteen values, at each overvoltage, is also plotted with a dashed red line.

Figure 6 shows the measured SPTR for each channel versus applied voltage in the SiPM array. A red, dashed line is also plotted, which shows the average SPTR over all channels. As has been previously reported (Kratochwil *et al* 2021), the best measured SPTR was found at overvoltage ≥*V*_br _+ 10 V, where SPTRs ranging from 99 to 150 ps were measured for the SiPM array and data acquisition. The best average SPTR was 117 ± 1 ps at 37 V, corresponding to *V*_br_ + 10 V. The average SPTR at the operating voltage for the remainder of the experimental studies (34 V, or *V*_br_ + 7 V) was 133 ± 1 ps (due to limitations in dynamic range of the data acquisition, as outlined in section 2.4).

### 3.2. Photon counting experiments

**Figure 7.**
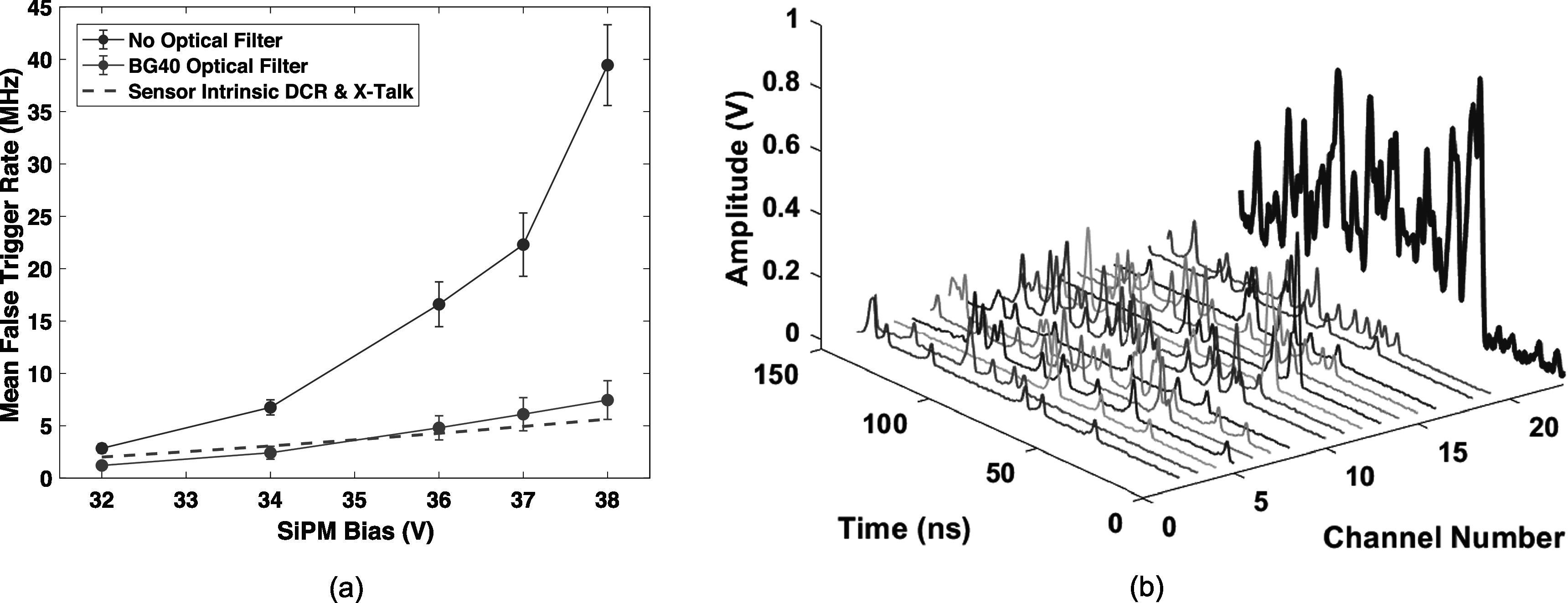
Reduction in optical photon noise enabled by the Schott BG40 optical glass filter is quantified in (a), where the optical bandpass virtually eliminates external optical crosstalk. Optical photon data streams from each channel of the prototype detector, for an example 511 keV photon interaction is shown in (b), along with the sum of all channels in blue, demonstrating the ability of the detector to generate temporal sparsity in the optical photon arrival times.

Figure 7(a) shows analysis performed to quantify the effect of the optical bandpass filter for reducing external optical crosstalk emissions, where the Ge-68 source was not present in the experiment, and a software trigger was used to digitize random 200 ns trace captures. With no radiation source present for these data, pulses represent dark counts, internal optical crosstalk, and any potential external optical crosstalk not absorbed by the bandpass filter. Counted pulses in these data thereby quantify the amount of ‘optical photon noise’ present in measurements with the radiation source (not resulting from detection of scintillation or Cherenkov photons), which is denoted in figure 7(a) as the average ‘mean false trigger rate’ (i.e. optical photon equivalent signal trigger rate due to uncorrelated and correlated noise) for a single pixel. Two measurements are shown with and without the optical glass filter in place. Without the bandpass filter, the mean false trigger rate increases drastically at higher overvoltage. However, with the optical filter coupled between the crystal and SiPM array, the mean false trigger rate trend for a single pixel follows a simple estimate of expected combined uncorrelated and correlated noise rate, derived from the SiPMs’ data sheet (dark count rate multiplied by crosstalk probability). Thus, the optical glass filter virtually eliminates external crosstalk in this detector configuration. With such a drastic reduction in correlated noise for a monolithic detector, sparsity in the arrival time profile of scintillation (and Cherenkov) light can be achieved in the SiPM array for 511 keV interactions, as shown in figure 7(b), where the optical photon stream for a single event is shown for each of the sixteen detector channels, and the sum of all channel responses is shown in blue.

**Figure 8.**
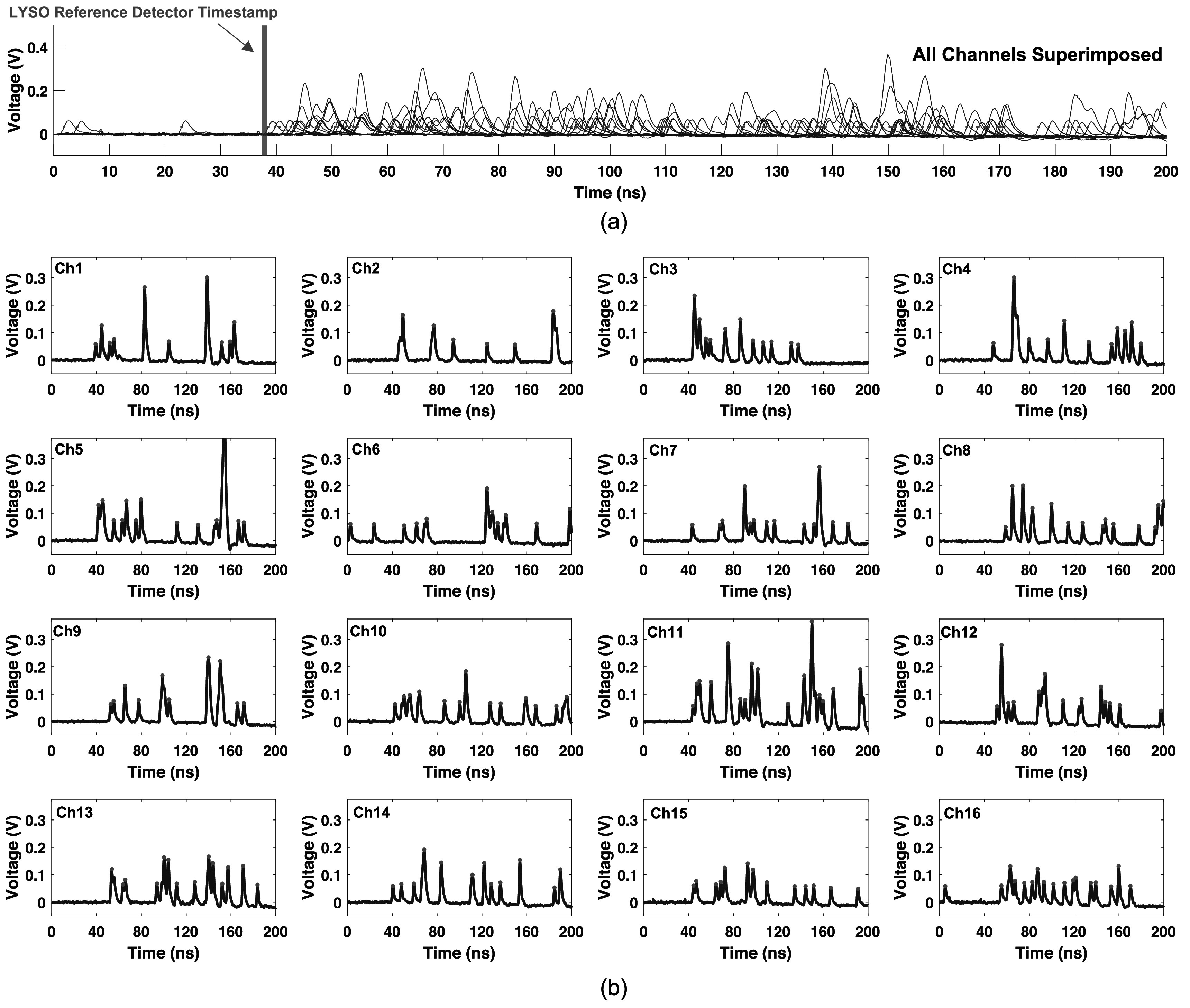
A detailed view of the scintillation photon counting procedure, for an example 511 keV photon interaction in the detector is shown. In (a), all channels are superimposed into a single plot, including a vertical red bar demarking the time pickoff from the LYSO:Ce reference detector. Red dots on the rising edge of each optical photon pulse denote time pickoff at half the single photon amplitude. Photon counting for each individual channel is shown in (b), where red dots indicate an optical photon pulse recorded by a simple peak finding algorithm.

To further demonstrate the photon counting capability of the prototype detector and experimental demonstration setup, figure 8 shows typical photon arrival time profiles for a randomly selected 511 keV photoelectric interaction. In figure 8(a), all channels are superimposed, with a red vertical line showing the timestamp from the LYSO:Ce reference detector. Red dots on the rising edge of the optical photon pulses indicate time pickoff for each pulse. The same arrival time profile, on a per-channel basis, is shown in figure 8(b), where red dots indicate peaks from the peak-finding-algorithm, which were used to perform photon counting. The average single photon amplitude for the photon counting measurements was 62 mV. The majority of optical photon pulses are single photons. In fact, 66% of all optical photon pulses were single photons, 21% had two optical photon equivalent amplitudes, and 13% had an amplitude equivalent to three or more photons. Due to the discrete nature of single photon amplitudes in SiPMs, the number of photons in each optical pulse (signal and noise) can be quantified, and every optical photon can be counted.

**Figure 9.**
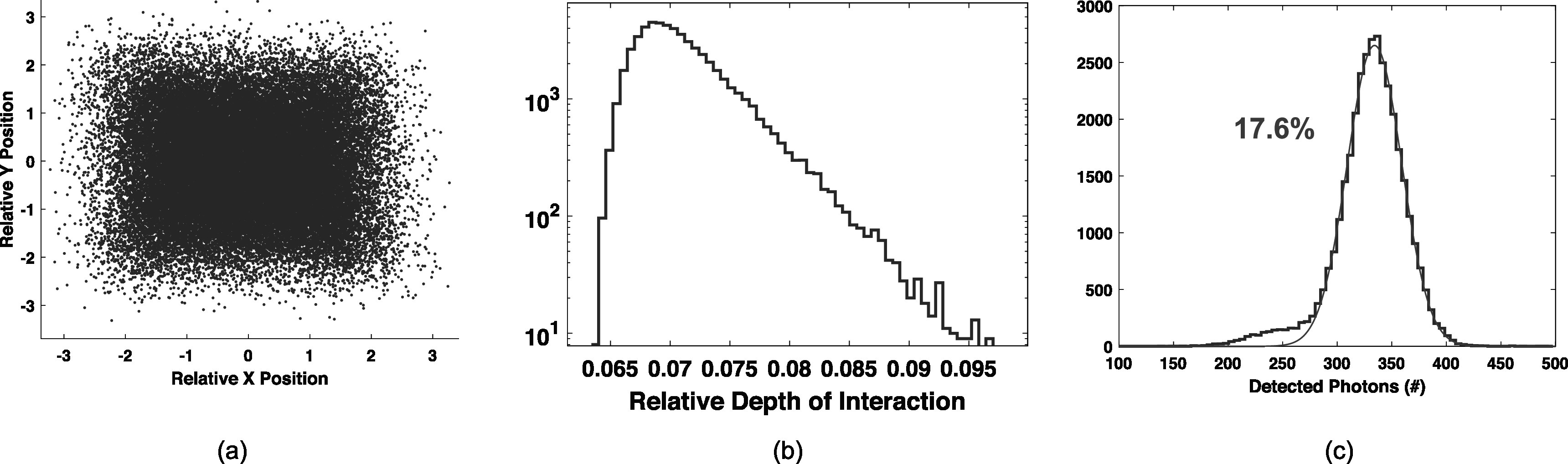
Relative X and Y positioning of events in the detector are shown in (a), along with relative depth distribution in (b). A 3D position of interaction corrected energy spectrum for the detector is shown in (c), where an energy resolution of 17.6% was achieved from a fit to the raw photon count distribution of 511 keV photoelectric interactions.

Figures 9(a) and (b) show relative x-y and depth positioning, respectively (units are arbitrary). The result of segmenting the crystal volume into 18 voxels and performing a light collection efficiency correction for each voxel, to correct a global energy spectrum taken from the sum of all counted photons for each event, is shown in figure 9(c). The achieved energy resolution was 17.6%, which was improved by 3.8% over a fit to the uncorrected global energy spectrum, at 18.1%.

**Figure 10.**
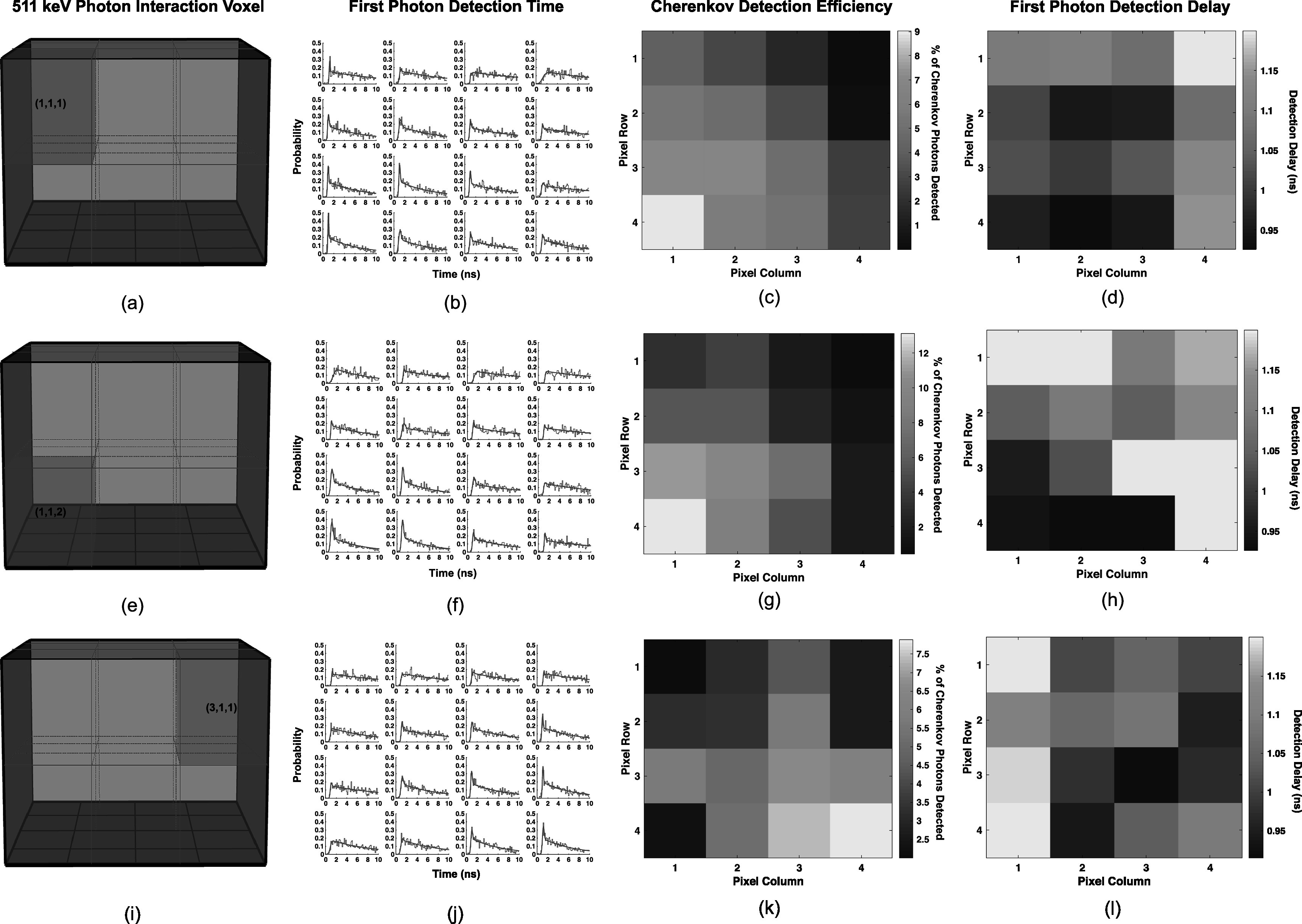
Examples of the 3D segmentation applied to the crystal volume, first detected photon distributions for each pixel for events generated in a single 3D voxel element, per-pixel percentage of detected Cherenkov photons, and per-pixel first photon delay are shown in (a)–(d). The same visualizations are presented for two additional 3D voxel elements in (e)–(g) and (i)–(l).

Figure 10 shows three examples of crystal segmentation implemented using the relative positioning information shown in figure 9, first photon arrival time distributions at each pixel for each interaction voxel, relative percentage of Cherenkov photons detected among pixels in the array for the interaction voxel, and time delay of the first detected photon for each scenario. The trends observed in photon arrival time distributions (figures 10(b), (f), and (j)) show higher Cherenkov photon detection in pixels immediately below the interaction voxel, and prompt distributions for interactions closer to the SiPM array (figure 10(f)) are broader due to large scintillation photon transit time jitter (larger disparity in forward and backward propagating photon arrival times). The relative Cherenkov detection efficiency (figures 10(c), (g), and (k)) and first photon arrival time delay (figures 10(d), (h), and (l)) also correlate with the arrival time distributions, where SiPM pixels closer to the interaction voxel exhibit higher relative Cherenkov detection efficiency and earlier first photon arrival times. These figures demonstrate that 3D position of interaction-dependent timing corrections can be derived by recording the arrival time profiles of optical photons with the prototype photon counting detector. The optical transit delay for each interaction voxel to each SiPM pixel was used to correct the timestamps for events in each interaction voxel and corresponding SiPM.

**Figure 11.**
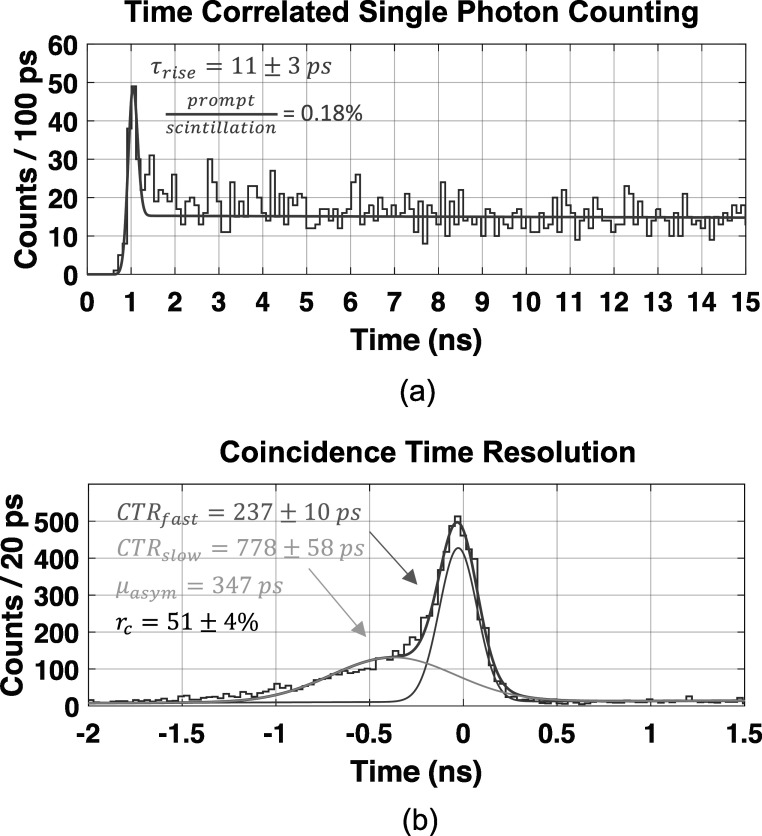
A time correlated single photon counting distribution is shown in (a), and a coincidence time resolution spectrum from the prototype demonstration setup is shown in (b).

Figure 11 shows two examples of time-based information from the photon counting detector. Specifically, a time correlated single photon counting spectrum in figure 11(a) and coincidence time distribution in figure 11(b). Figure 11(a) was produced by randomly selecting timestamps (after arrival time delay correction) for the SiPM with the best measured SPTR, for each event within the ∼150 ns data acquisition capture window and applying equation (3) (same one for arrival time distributions) to the observed distribution. Interestingly, the observed rise time for the bi-exponential component of the model is similar to that reported for time correlated single photon counting experiments with BGO (Gundacker *et al* 2020). Although the capture window of our experimental setup is not long enough to make a precise determination of the long time component of the distribution, error on the fitted value includes the long component also reported for BGO (365 ns, as listed in table 1). When calculating the integral of the fitted prompt and bi-exponential distributions over 1 microsecond and correcting for photon detection efficiency (shown in table 1), the estimated number of Cherenkov photons produced is 18.5, which is also similar with other empirically derived estimates of Cherenkov yield for BGO (Gundacker *et al* 2020). This figure demonstrates an interesting capability of the photon counting detector prototype, and the agreement of parameters extracted from a fit to the distribution with other works gives further confidence in its performance and capabilities.

The coincidence time distribution shown in figure 11(b) is comprised of two primary components, one fast and another slow, representing events where timestamps were derived from Cherenkov or scintillation photons, respectively (Kratochwil *et al* 2020). The resulting CTR, versus our reference detector, for the fast distribution was 237 ± 10 ps FWHM, and 778 ± 58 ps FWHM was observed for the slow distribution (with a 347 ps asymmetric offset, *μ*_*asym*_). The percentage of events comprising the fast distribution (*r*_*c*_) was 51 ± 4%. The expected CTR between two identical prototype detectors, after subtracting the influence of the reference detector, would be 315 ps and 1.09 ns for the fast and slow distributions.

## 4. Discussion

We have presented a first-ever demonstration of a scintillation photon counting detector prototype with analog SiPMs. The overall concept for our detector is to spread scintillation photons over a large SiPM array with a monolithic scintillation crystal. If the scintillator type and geometry and number of SiPM channels are appropriately configured, temporal sparsity in the arrival time of optical photons, at each detector channel, can be achieved. If this sparsity is greater than the FWHM of the single photon response shape of the SiPM array’s electronic readout, each optical photon may, in principle, be resolved. To investigate this new detector concept, we constructed a prototype demonstration setup with a monolithic BGO scintillation crystal, sixteen-channel SiPM array, a 1 mm thick optical bandpass glass to virtually eliminate external optical crosstalk from the SiPM array, multichannel LNHF electronic readout, and a custom-calibrated, fast, multichannel digitizer. Interpretation of our findings with this prototype detector is detailed in the following sections.

### 4.1. Impact of optical bandpass filter

The impact of the optical bandpass filter on ‘optical photon noise’ present in our detector design was shown in figure 7(a). Counting dark counts and optical crosstalk photons observed in our prototype detector with no radiation source present, using a random trigger, with and without the optical bandpass filter coupled between the BGO crystal and SiPM array allowed us to quantify equivalent ‘optical photon noise’ (i.e. not scintillation or Cherenkov light) present during measurements. Observed statistics within our 200 ns capture window were converted to a mean noise event rate for each detector pixel, as a function of applied overvoltage, which we denoted as a ‘mean false trigger rate’. The observed mean false trigger rate per-pixel, with no bandpass filter in place, increased dramatically at higher overvoltage, towards 40 MHz at *V*_br_ + 12 V. With the glass filter in place, the mean false trigger rate increased linearly with applied overvoltage. In fact, the total mean false trigger rate matched the expected combination of dark count rate with internal optical crosstalk probability detailed on the AFBR-S4N33C0133 data sheet. Thus, the optical bandpass filter virtually eliminates the contribution of external optical crosstalk from our measurements. This is key to realizing our detector concept in an implementation that can be operated at room temperature and high overvoltage, thus being tractable to larger scale implementations and not sacrificing in performance to avoid the influence of external optical crosstalk. We also note that other works have suggested the use of optical bandpass filters to reduce the magnitude of optical crosstalk from SiPM arrays (Barton *et al* 2009, Masuda *et al* 2021). In the present work, we apply this approach, for the first time, with a monolithic scintillation detector.

### 4.2. Photon counting capability of the detector design

In section 2.1, we presented a calculation for the approximate single photon response shape and SiPM array size for two example scintillation detectors. These estimations are only meant to aid in explanation of the detector concept and provide a starting point for the selection of a detector readout configuration. These estimates are provided for detectors with characteristics outlined in table 1 and do not account for the unavoidable fact that some fraction of 511 keV photon interactions will interact close to the photosensor, which will not provide adequate light spread across the sensor array. In our experimental data, we found that 66% of optical photon pulses were single photons, 21% comprised two photons, and 13% represented three or more photons. Thus, the majority of detected optical photon pulses are single light photons which can be uniquely counted.

There are two primary points of discussion about the observed photon counting statistics. First, as discussed in section 4.1 above, the optical bandpass filter virtually eliminates external optical crosstalk from our measurements, but internal optical crosstalk (optical photon crosstalk between cells in an SiPM pixel) remains present. Thus, some percentage of the two-or-more optical photon pulses (34% of all detected optical photon pulses) are the result of internal optical crosstalk, as opposed to the pileup of scintillation/Cherenkov photons in the photosensor array. The two-photon pulses occur with a probability less than crosstalk probability listed on the AFBR-S4N33C013 SiPM datasheet (35%). This is expected with the optical filter in place, as measurements presented in the sensor's datasheet include crosstalk photons that reflect at the glass window-air interface and contribute to that characterization (Masuda *et al* 2021), which are filtered in our design. It could be that the majority of two-or-more optical photon pulses are the result of internal crosstalk, but that cannot be directly quantified from our data. Ongoing studies will more precisely disentangle the population of two-or-more-photon pulses which represent scintillation pile-up versus correlated noise detection, which will be presented in future work. However, a second major point on this subject is that even the two-or-more-photon pulses allow each detected optical photon to be counted, due to the discrete nature of single photon voltage pulse amplitude. The ability to create a unique timestamp for each of the optical photons is lost, but a timestamp to represent the arrival time of all the photons contained in that pulse or ‘bunch’ can be derived from leading edge discrimination on the pulse's rising edge. Thus, each photon can still be counted, and every single photon and photon ‘bunch’ has a unique timestamp.

### 4.3. Single photon time resolution of the detector and experimental setup

We presented SPTR for each detector channel as a function of applied overvoltage in figure 6. The measured values are significantly worse than what has been demonstrated with the same SiPMs, where sub-100 ps has been demonstrated (Cates and Choong 2022) across a wide operating voltage range. There are two primary reasons for this. First, the gain of the first RF amplifier in the signal processing chain, the Minicircuits RAM-8SM+ shown in figure 2(a), had to be reduced by lowering applied voltage in order to fit the detector's raw waveform within the 1 V dynamic range of our CAEN V1742 digitizer. This inherently limits the frequency response of the device, meaning the circuit element is band-limiting the signals below <1100 MHz. For these LNHF electronic readout topologies, ≥1500 MHz bandwidth should be maintained for optimal performance, as discussed in (Gundacker *et al* 2019). Furthermore, the front-end buffers of the CAEN V1742 digitizer band-limit the signal to ≤500 MHz. The impact of these elements on rising edge slew increases the influence of electronic noise and results in higher observed SPTR. Nonetheless, the 133 ps FWHM average SPTR value achieved for our detector at the operating voltage employed in our coincidence experiments was sufficient for these first prototype demonstration studies.

### 4.4. Coincidence experiments with the prototype demonstration setup

We employed the experimental setup shown in figure 4 to demonstrate the use of counted photon statistics in 3D positioning of 511 keV photon interactions, estimating event energy, integrating 3D-interaction-dependent data corrections for energy and timing estimators, time correlated photon counting capabilities, and achievable coincidence time resolution with the prototype detector. In this work, we used simple analytical methods for estimating relative 3D position of interaction within the crystal volume. This approach is limited in accuracy, as absolute positioning requires calibrations from pencil- or fan-beam irradiation across the detector area (Borghi *et al* 2016a), or other approaches can be used (Gonzalez-Montoro *et al* 2021). This is especially true for the relatively high aspect ratio of our crystal geometry, which introduces significant bias and nonlinearity near the crystal's edges with these analytical estimates for relative position of interaction. It is beyond the scope of the present work to absolutely calibrate position of interaction and quantify this performance metric, as our primary aim is to demonstrate the photon counting detector concept with analog SiPMs. In our data analysis, we uniformly segmented events across the area of the detector and segmented relative depth of interaction values according to the linear attenuation coefficient for BGO. The crystal volume was segmented into relatively large voxels to account for this nonlinearity, effectively separating the detector into ‘edge’ and ‘center’ voxels, each with two ‘depth’ bins. Even this coarse voxelization showed the ability to correct for variations in light collection efficiency throughout the detector volume, which translated to a marginal improvement in energy resolution of 3.8%.

Inspired by previous works that implemented first detected photon time information at each pixel, for each 3D position of interaction voxel, into maximum likelihood-based estimators for time of interaction (van Dam *et al* 2013, Tabacchini *et al* 2015), we applied a modified first photon detection time delay correction to our detected photon timestamps. Since our first photon detection time distributions include Cherenkov light, we included a term to characterize these prompt signatures in the arrival time distributions, as depicted in figure 5. The arrival time distributions for three different voxels were shown in figure 10, which clearly demonstrate unique arrival time distributions for each voxel. Moreover, the shape of these distributions match intuition, based on light transport in the crystal volume. SiPMs directly below each voxel have the highest percentage of detected Cherenkov photons and the shortest delay time, and moving closer to the sensor further drives these values higher and lower, respectively. Delay distributions for interactions at the top of the crystal also exhibit sharper prompt contributions, where scintillation photon transit time variance is lower, due to events occurring near the top of the crystal. While we only extracted a single delay parameter for each pixel-voxel combination to perform a time delay skew correction, it is clear that the photon counting detector's output is data-rich. The prompt Cherenkov signature in the delay distributions also provides a direct measurement of optical transit time in the crystal not influenced by scintillation kinetics. Thus, there is likely the opportunity for existing or new approaches using advanced time of interaction estimators to accurately account for scintillation transit time skew in the crystal volume. For detection media exhibiting prompt optical signatures, such as Cherenkov light, this could potentially be a pathway for large area, high-sensitivity time-of-flight PET detector performance limited primarily by SPTR, a device characteristic which can be improved towards 10's of ps (Gundacker *et al* 2023). More advanced estimators, like those in (van Dam *et al* 2013, Tabacchini *et al* 2015) and machine learning based approaches will be the subject of future studies.

The ability of our prototype detector to perform time correlated single photon counting measurements was demonstrated in figure 11(a) by randomly selecting single photon events after 3D interaction dependent skew correction, for a single pixel. While our prototype demonstrator's SPTR is not ideal for this measurement, it is interesting that the observed rise time for BGO and fraction of Cherenkov light are similar to other measurements of these parameters (Gundacker *et al* 2020). The 200 ns capture window of the DRS4-based digitizer used in this work prohibited accurate quantization of the decay time parameter. We present this result as a preliminary demonstration of this potential new capability. Typical time correlated single photon counting techniques for measuring scintillation kinetics rely on greatly reducing the probability of single photon detection from the sample in order to remove the influence of bias in the measurement. A consequence of this condition is that the measurement times can be quite long. Fast pulsed x-ray measurements can be employed for fast measurements of scintillation rise and decay, but these may not provide sufficient energy deposition to investigate prompt signatures, such as Cherenkov light. A scintillation photon counting detector similar to what we have presented could potentially be used for such characterization. However, further studies are required to capture the full timing envelope of a material and more thoroughly investigate this capability, which is beyond the scope of the present work. In this work, the time correlated single photon counting spectrum in figure 11(a), provides another example of the prototype's ability to perform scintillation/Cherenkov photon counting and the kind of data which can be extracted from its output.

The CTR of our experimental setup is shown in figure 11(b), where 237 ± 10 ps FWHM was observed for the fast component of the multicomponent fit to the time delay distribution. Considering this was generated in coincidence with a small, fast reference detector (81 ps SDTR), the observed CTR is not representative of state-of-the-art for long crystal elements of BGO. This is due to the limitations on achievable SPTR outlined in section 4.3 and the required low SiPM operating voltage (*V*_br _+ 7 V), well below the optimal set point for timing measurements with AFBR-S4N33C0133 SiPMs (∼*V*_br _+ 11–*V*_br_ + 12 V).

Despite limitations the prototype demonstration detector and experimental setup impose on achievable CTR, there is significant promise for this detector concept with BGO specifically. In Gundacker *et al* (2020), Monte Carlo simulations predicted that a detector which combines BGO with a UV-sensitive photosensor capable of time pickoff from the first detected photon with excellent SPTR can greatly outperform leading time pickoff of an aggregate pulse. Studies showed CTR approaching ∼150 ps may be possible for 20 mm length crystals and SPTR achievable with current commercial devices. Using data corrections like those employed in this work, or more advanced implementations, it may be possible to reduce this even further, towards sub-100 ps CTR predicted for smaller crystal geometries, thereby providing candidate TOF-PET detectors with ultraprecise timing performance and high 511 keV photon detection efficiency.

### 4.5. Translation of detector concept into a tractable architecture

**Figure 12.**
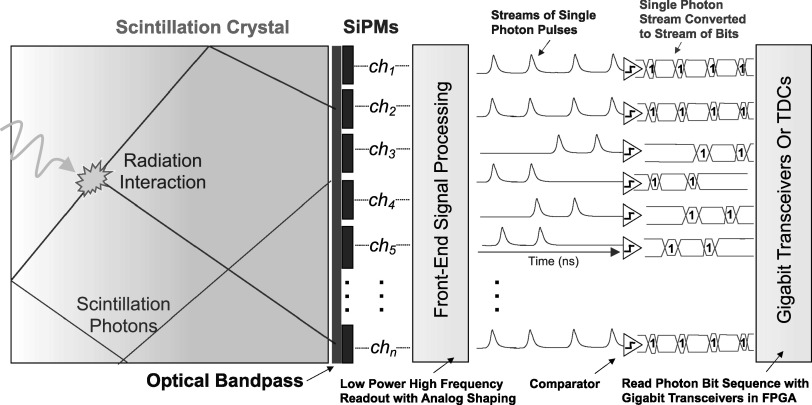
A tractable implementation of our photon counting detector concept is illustrated.

As a last point of discussion, we acknowledge that our proof-of-concept demonstration setup is limited in translation, in the exact embodiment presented in section 2.1. However, we have also conceived of a tractable detector design and electronic readout topology to realize this detector concept for advanced PET imaging systems. Figure 12 shows an illustration of a scalable implementation of our photon detector concept. The key difference between this design and the prototype presented in this work is in the method for event digitization. Creating sparsity in arrival time profiles of scintillation light at each detector element allows for streams of optical photons to be digitized as streams of ‘bits’ with a comparator. If the resulting digital output is simply treated as data, it can be directly processed by gigabit transceivers, which are now available in very high speeds and density in modern field programmable gate arrays (FPGAs). In this way, the number of bits in each ‘bitstream’ corresponds to the number of detected photons, and the bit position within a data word corresponds to time of arrival, within an event capture. For the case of optical photon pile-up, the number of photons in each bunch is still available from time-over-threshold information, and timestamps for each photon in a bunch from the rising edge. also note that one could alternatively employ multi-channel TDCs, if only the first detected photon information is required for a particular application. For this detector configuration, we also aim to use a low power implementation of the LNHF readout (Cates and Choong 2022) for each channel, with integrated analog shaping to produce tight semi-Gaussian pulse shapes, which together can provide the necessary single photon response shapes with excellent SPTR, at relatively low power consumption per channel (∼10 mW).

## 5. Conclusions

We have presented a first-ever demonstration of a prototype scintillation photon counting detector concept, comprising all off-the-shelf-components, including analog SiPMs. The experimental setup designed for this prototype showed the ability to count and provide unique timestamps for 66% of all optical photons from a monolithic BGO scintillator. The remaining 34% of two-or-more-photon pulses are also independently counted, but each photon bunch shares a common timestamp. The setup showed good SPTR for 3 × 3 mm^2^ AFBR-S4N33C013 SiPMs (117 ps at *V*_br_ + 10 V), 3D event positioning, and the ability to implement position-of-interaction-dependent data corrections on event energy and time of interaction estimators, achieving 17.6% energy resolution and 237 ± 10 ps FWHM CTR (fast spectral component) versus a reference detector. This detector concept presents a promising design for large area, high sensitivity TOF-PET detector modules that can implement advanced event positioning and time of interaction estimators, which could push state-of-the-art performance.

## Data Availability

All data that support the findings of this study are included within the article (and any supplementary information files).
